# Timing of respiratory syncytial virus and influenza epidemic activity in five regions of Argentina, 2007‐2016

**DOI:** 10.1111/irv.12596

**Published:** 2018-11-20

**Authors:** Elsa Baumeister, Jazmin Duque, Teresa Varela, Rakhee Palekar, Paula Couto, Vilma Savy, Carlos Giovacchini, Amber K. Haynes, Brian Rha, Carmen S. Arriola, Susan I. Gerber, Eduardo Azziz–Baumgartner

**Affiliations:** ^1^ Servicio Virosis Respiratorias Instituto Nacional de Enfermedades Infecciosas INEI–ANLIS “Dr. Carlos G. Malbrán” Buenos Aires Argentina; ^2^ Influenza Division National Center for Immunization and Respiratory Diseases U.S. Centers for Disease Control and Prevention Atlanta Georgia; ^3^ Battelle Atlanta Atlanta Georgia; ^4^ Sistema Nacional de Vigilancia por Laboratorio Sistema Nacional de Vigilancia de la Salud Ministerio de Salud de la Nación Buenos Aires Argentina; ^5^ Pan American Health Organization Washington District of Columbia; ^6^ Division of Viral Diseases U.S. Centers for Disease Control and Prevention Atlanta Georgia; ^7^ U.S. Public Health Service Rockville Maryland

**Keywords:** Argentina, epidemic, flu, influenza, respiratory syncytial, seasonality

## Abstract

**Background:**

Within‐country differences in the timing of RSV and influenza epidemics have not been assessed in Argentina, the eighth largest country in the world by area.

**Objective:**

We aimed to compare seasonality for RSV and influenza both nationally and in each of the five regions to inform Argentina’s prevention and treatment guidelines.

**Method:**

The Argentine National Laboratories and Health Institutes Administration collected respiratory specimens from clinical practices, outbreak investigations, and respiratory virus surveillance in 2007‐2016; these were tested using immunofluorescence or RT‐PCR techniques. We calculated weekly percent positive (PP) and defined season onset as >2 consecutive weeks when PP exceeded the annual mean for the respective year and region. Median season measures (onset, offset and peak) and the established mean method were calculated for each virus.

**Results:**

An annual median 59 396 specimens were tested for RSV and 60 931 for influenza; 21–29% tested positive for RSV and 2–7% for influenza. National RSV activity began in April; region‐specific start weeks varied by 7 weeks. Duration of RSV activity did not vary widely by region (16–18 weeks in duration). National influenza activity started in June; region‐specific start weeks varied by 3 weeks. Duration of influenza epidemic activity varied more by region than that of RSV (7–13 weeks in duration).

**Conclusion:**

In Argentina, RSV and influenza activity overlapped during the winter months. RSV season tended to begin prior to the influenza season, and showed more variation in start week by region. Influenza seasons tended to vary more in duration than RSV seasons.

## 1 INTRODUCTION

Respiratory syncytial virus (RSV) and influenza viruses cause substantial disease and economic burden.^1^, ^2^ Surveillance systems in temperate countries demonstrate that RSV and influenza activity are seasonal and typically peak during colder months.^3^, ^4^, ^5^, ^6^ In large countries like China and India, regional differences in respiratory virus activity can be substantial.^7^, ^8^ These differences can be large enough that public health officials recommend administering different influenza vaccine formulations at different times of the year within the same country to mitigate seasonal activity.^9^, ^10^ Regional differences in seasonal activity might also affect when persons at high risk of influenza‐related complications are empirically treated with antivirals^11^ and when RSV immunoprophylaxis is administered.^12^ Optimizing the timing of these interventions may increase national coverage and cost‐effectiveness.^6^, ^7^, ^8^, ^13^, ^14^ It is therefore useful to establish the timing of RSV and influenza seasons to inform providers when they should anticipate administering virus‐specific interventions.

Argentina's prevention and treatment guidelines for RSV and influenza are provided at the national level. Starting in 2015, Argentina's Pediatric Society recommended the use of palivizumab, a monoclonal antibody, as a cost‐effective method to prevent severe RSV illness among Argentinian children at high risk of hospitalizations due to RSV infection.^15^ Specifically, the Society recommended that clinicians administer five doses of palivizumab (15 mg/kg) every 30 days during April‐September for children aged ≤2 years with prematurity, congenital heart disease, or bronchopulmonary dysplasia.^16^ Rodriguez et al^17^ found that treating ~5 high‐risk patients with palivizumab averted one RSV‐associated hospitalization.

In addition, the Argentina Ministry of Health (MOH) recommends influenza vaccination for all persons aged >6 months and especially for those at high risk of influenza illness complications like pregnant women and very young children.^18^ Influenza vaccination starts as early as March in Buenos Aires, Argentina's capital and largest city, and continues throughout the influenza season. Influenza vaccines have been shown to provide moderate protection against influenza‐associated hospitalizations in young children and older adults.^19^ In Argentina, vaccine coverage among young children has reached 80%^20^ and about half of adults report getting vaccinated against influenza.^21^ The MOH also recommends the use of empiric oseltamivir among persons at high risk of influenza illness complications and among hospitalized persons suspected of having influenza throughout the season.^22^

In this study, we characterize RSV and influenza seasonality in five regions of Argentina: northwest, northeast, central, cuyo (central west), and south. Neighboring provinces share similar geoclimatic settings and work together as regions for administrative purposes. Awareness of the regional and annual timing of these seasons might help policymakers and clinicians better time annual subnational RSV and influenza prevention and control efforts.

## 2 METHODS

### 2.1 Data source

The design and methods for the Argentine World Health Organization's (WHO) National Influenza Center (NIC) has been described previously.^23^ Respiratory specimens submitted for influenza and other respiratory virus testing from healthcare settings located across Argentina's 23 provinces are submitted weekly to Argentina's National Laboratories and Health Institutes Administration (ANLIS). Argentina's Buenos Aires NIC participates in WHO's Global Influenza Surveillance and Response System and helps coordinate ANLIS. ANLIS conducts respiratory virus surveillance and year‐round virologic testing in coordination with 65 laboratories throughout the country and more than 7000 healthcare sites. Currently, each healthcare site collects nasal aspirates, throat, and/or nasal swabs from children and adults suspected of respiratory illness. A subset of participating facilities use the WHO's case definitions for influenza‐like illness (ILI) and severe acute respiratory illness (SARI) to guide respiratory testing while other facilities test based on physician ordering practices and outbreak response.^24^ ANLIS collects data on patient age and clinical setting (inpatient vs outpatient). The specimens are tested by indirect immunofluorescence (IFA) assays for RSV. Until 2009, IFA was used to test for influenza A and B, but as of 2010, all influenza testing was carried out through real‐time reverse transcription polymerase chain reaction (rRT‐PCR). The Buenos Aires NIC uses the influenza diagnosis protocol developed by the U.S. Centers for Disease Control for rRT‐PCR tests.^17^, ^18^

### 2.2 Descriptive data analyses

Specimen test results from Argentina's 23 provinces were grouped into five administrative regions: northwest, northeast, central, cuyo (central west), and south. To account for age differences in risk of RSV and influenza infection, frequency and proportion of RSV and influenza‐positive tests detected from 2007 to 2016 were calculated for the following age‐groups: <1, 1‐5, 5‐14, 15‐64, and <64 years.

To describe annual virus activity at the national and regional levels, the following parameters were first determined for each calendar year for each virus: the mean percent positive for the year, and the season start, peak, and end. We defined the season start for a given calendar year as the week when the proportion of specimens testing positive for a specific virus was greater than the annual mean percent positive for two or more consecutive weeks over a 1‐year period. The season ended when the proportion of positive specimens for a specific virus fell below the annual mean for two or more consecutive weeks. The peak was defined as the week with the maximum weekly proportion of positive specimens that occurred between the start and end of the season. Having defined the virus annual activity for each year, we applied a median method to calculate summary virus activity for the 10‐year period by taking the median (and interquartile range) of the annual activity start, peak, and end week values over the 10 years. These calculations were performed at the national level and for each region.

Because separate published “mean methods” have been previously described and validated to assess multiannual influenza circulation,^5^ an additional mean method was applied to specimens tested for RSV and influenza to compare the circulations of RSV and influenza in Argentina from 2007 to 2016 described herein. The sum of viral detections and specimens tested for a given virus was calculated for each epidemic week across 10 years to determine 52 weekly percent positive values for the nation and each region. The 10‐year onset was the first of two consecutive epidemic weeks when the weekly percent positive was greater than the 10‐year weekly mean. The 10‐year offset was the last of two consecutive epidemic weeks when the 10‐year weekly percent positive exceeded the 10‐year mean.^25^ The 10‐year peak was the 10‐year week with the highest weekly percent positive. Descriptive and quantitative analyses were performed using sas 9.3 and Microsoft Office Excel 2016.

### 2.3 Ethical considerations

The deidentified data used in this study were collected via routine clinical and public health functions; their analysis was part of a program evaluation and not considered human subjects research.

## 3 RESULTS

From January 2007 through December 2016, Argentina's ANLIS tested an annual median of 59 396 respiratory specimens (interquartile range [IQR] 28 487‐68 109) for RSV and 60 931 (IQR 29 690‐71 041) for influenza. Of the 496 789 specimens tested for RSV, 24% (n = 118 329) were positive, and of the 520 116 specimens tested for influenza, 5% were positive (n = 24 700). Of the specimens tested for RSV, 64% were collected from the central region, 95% were collected at an inpatient setting, 49% were from children <1 year, and 24% were collected from children aged 1‐5 years. Of the specimens tested for influenza, 65% were collected from the central region, 94% were collected at an inpatient setting, 47% were collected from children aged <1 year, and 23% were collected from children aged 1‐5 years (Table 1).

**Table 1:** Respiratory specimens tested in Argentina's National Laboratories and Health Institutes Administration for respiratory syncytial virus and influenza, 2007‐2016

	Respiratory syncytial virus (N = 496 789)	Influenza (N = 520 116)	Population^a^
	Number of samples tested (column %)	Number of samples tested (column %)	
Year			
2007	11 877 (2)	11 583 (2)	–
2008	12 755 (3)	10 978 (2)	–
2009	48 659 (10)	47 992 (9)	–
2010	54 773 (11)	54 483 (10)	–
2011	21 763 (4)	23 589 (5)	–
2012	69 383 (14)	72 251 (14)	–
2013	74 663 (15)	82 068 (16)	–
2014	64 019 (13)	67 413 (13)	–
2015	64 287 (13)	67 378 (13)	–
2016	74 610 (15)	82 381 (16)	–
Age‐group (y)			
<1	244 010 (49)	243 099 (47)	–
1‐5	117 352 (24)	118 638 (23)	–
5‐14	31 496 (6)	32 962 (6)	–
15‐64	27 653 (6)	36 730 (7)	–
>64	7651 (2)	10 262 (2)	–
Missing	68 627 (14)	78 425 (15)	–
Region			
Northwest	60 247 (12)	59 358 (11)	4 911 412
Northeast	36 678 (7)	37 927 (7)	3 679 609
Central	319 033 (64)	339 430 (65)	26 254 642
Cuyo	40 520 (8)	38 800 (7)	2 852 294
South	39 077 (8)	44 601 (9)	2 419 139
Setting			
Inpatient	474 288 (95)	491 128 (94)	–
Outpatient	22 501 (5)	28 988 (6)	–

a Censo Nacional de Población, Hogares y Viviendas 2010, Instituto Nacional de Estadística y Censos, República Argentina.

In general, the median 10‐year method resulted in a national RSV season that occurred during April‐August (week 17‐week 34), during which 21%‐29% of respiratory specimens tested for RSV were positive (Table 2). At the regional level, the overall 10‐year median start week for annual activity varied by region from April to June (week 16‐week 23). The RSV annual activity tended to start 7 weeks earlier in northwest Argentina (median start week 16 [IQR 10‐19]) than in southern Argentina (median start week 23 [IQR 22‐23]). Nationally, the RSV annual activity typically peaked in June (median peak week 25 [IQR: 23‐26]), when the weekly percent positive was 40%. Across regions, RSV annual activity tended to peak 6 weeks earlier in northwest Argentina in June (median peak week 23; IQR 21‐24) compared to the typical July peak in southern Argentina (median peak week 29; IQR 28‐32). RSV season duration tended to range from 16 to 18 weeks in length (Table 2).

**Table 2:** Respiratory syncytial virus (RSV) and influenza epidemic periods in Argentina and its subregions, National Laboratories and Health Institutes Administration, 2007‐2016

Subregion (climate)	RSV			Influenza		
	Median week and month (IQR)			Median week and month (IQR)		
	Start	Peak	End	Start	Peak	End
Northeast^a^^,^ b (mix of semiarid and tropical)	18 Apr (14‐21)	29 Jul (27‐30)	36 Sep (33‐39)	28 Jul (24‐32)	34 Aug (29‐41)	37 Sep (36‐39)
Northwest^c^ (arid and subtropical)	16 Apr (10‐19)	23 Jun (21‐24)	32 Aug (30‐35)	28 Jul (25‐29)	34 Aug (29‐40)	41 Oct (37‐43)
Central (semiarid and temperate)	18 Apr (16‐18)	24 Jun (23‐25)	34 Aug (32‐34)	25 Jun (21‐27)	29 Jul (24‐35)	35 Aug (30‐39)
Cuyo^c^^,^ d (semiarid and temperate)	22 May (20‐22)	30 Jul (27‐31)	39 Sep (36‐41)	26 Jun (24‐30)	33 Aug (28‐37)	33 Aug (31‐42)
South^a^^,^ b (mix of arid/semiarid and extreme precipitation and temperate/cool temperate)	23 Jun (22‐23)	29 Jul (28‐32)	41 Oct (39‐44)	28 Jul (25‐32)	32 Aug (26‐38)	40 Oct (32‐45)
National	17 Apr (15‐17)	25 Jun (23‐26)	34 Aug (34‐35)	25 Jun (21‐29)	30 Jul (26‐37)	40 Oct (33‐44)

IQR, interquartile range.

a An 8‐y median is shown. Influenza data were insufficient to determine annual activity for 2007 and 2008.

b An 8‐y median is shown. Respiratory syncytial virus data were insufficient to determine annual activity for 2007 and 2008.

c A 9‐y median is shown. Influenza data were insufficient to determine annual activity for 2007.

d A 9‐y median is shown. Respiratory syncytial virus data were insufficient to determine annual activity for 2007.

Influenza seasons typically occurred during June to October (week 25‐week 40), during which 3%‐7% of specimens tested for influenza were positive. Region‐to‐region, the median start week of each season ranged from June to July (week 25‐week 28). Influenza annual activity only started 3 weeks earlier in central Argentina (June, week 25 [IQR 21‐27]) compared to northeast (July, week 28 [IQR 24‐32]), northwest (July, week 28 [IQR 25‐29]), and south Argentina (July, week 28 [IQR 25‐32]). Nationally, influenza peaked in July (week 30 [IQR: week 26‐week 37]), when the weekly percentage of influenza‐positive specimens was 5%. The average influenza season peaked 5 weeks earlier in central Argentina (July, week 29 [IQR 24‐35]) when compared to the northeast (August, week 34 [IQR 29‐41]) and the northwest (August, week 34 [IQR 29‐40]). Across regions, influenza seasons lasted 7‐13 weeks in duration (Table 2).

**Figure 1:**
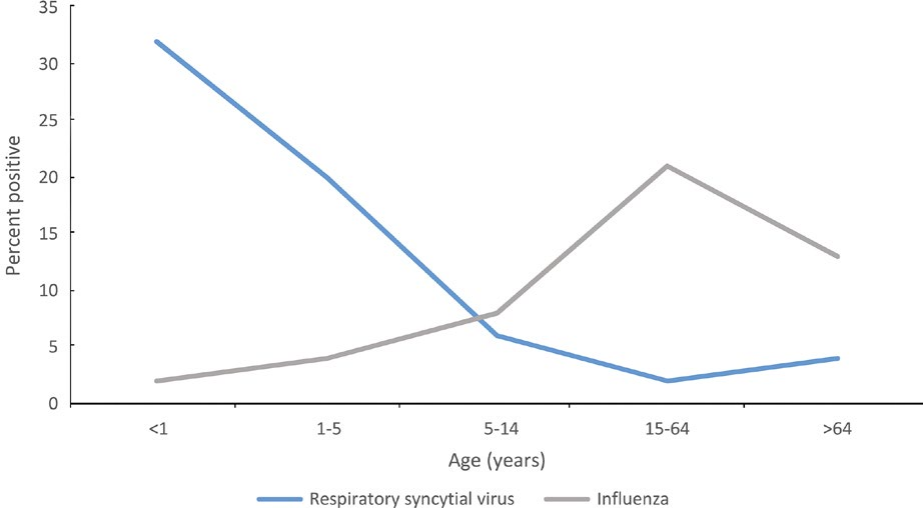
Age distribution of respiratory specimens tested for respiratory syncytial virus and influenza, Argentina's National Laboratories and Health Institutes Administration, 2007‐2016

Among RSV‐tested specimens, RSV positivity was highest among children aged <1 year (32%; n = 76 883) and aged 1‐5 years (20%; n = 23 596) compared to any other age‐group (*P* < 0.001) regardless of patient setting (Figure 1). Age data were missing for 68 627 (14%) specimens tested for RSV, of which 14 905 (22%) tested positive for the virus. In contrast, influenza positivity was highest among persons aged 15‐64 years (*P* < 0.001; Figure 1). Age data were missing for 78 425 (15%) specimens tested for influenza, of which 3536 (5%) tested positive for the influenza virus.

**Figure 2:**
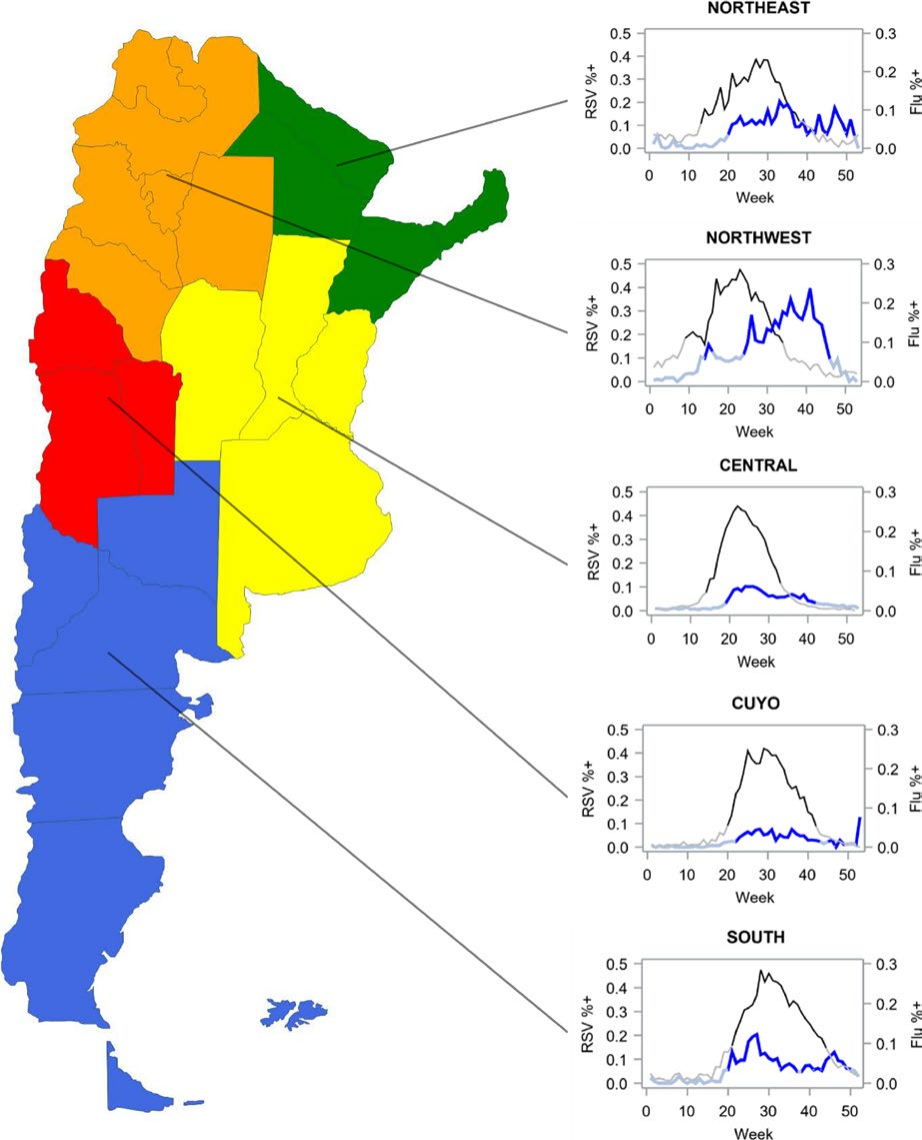
Weekly regional respiratory syncytial virus and influenza activity according to the mean method, Argentina's National Laboratories and Health Institutes Administration, 2007‐2016*

According to the mean method, the national RSV activity peaked before the influenza season (Figure 2). On average, RSV activity started in April (week 17) and influenza activity started 8 weeks later in June (week 25; Figure 2). The largest difference between the start of RSV seasons and the start of influenza seasons (12 weeks) was observed in the northwest part of the country.

Examination of the defined annual virus seasons for both RSV and influenza revealed that the timing of virus activity in central Argentina in any given year was similar to the national season average. Regional differences in RSV activity were noted each year (Figure S1), with season start weeks that varied by 7 weeks, starting as early as April in the northwest Argentina and as late as June in southern Argentina (Figure 2). RSV onset and offset across regions tended to follow similar timing, such that the duration of virus activity did not vary widely by region (duration of 16‐18 weeks). National influenza activity, however, frequently began in June (onset ranging from week 13 to week 38) (Figure S2) and duration varied by region (Figure 2). The 2009 and 2010 influenza epidemics, when influenza A (H1N1) pdm09 virus was first identified, occurred during the months when seasonal influenza epidemics typically occur (Figure S2).

## 4 DISCUSSION

Our analysis of Argentinian surveillance data from 2007 to 2016 indicates that RSV and influenza cocirculated during the winter months, as has been observed in other temperate climates.^26^ However, RSV seasons tended to precede influenza seasons at the national and regional levels during a 10‐year period, and for most of the individual study years assessed. In addition, the timing of RSV seasons varied across Argentina's five geographically diverse administrative regions. For example, the median start of the annual activities differed by an average of 7 weeks between the northwest and south regions of Argentina. In contrast, there was little variation in the median start of influenza activity throughout Argentina's region. However, RSV season duration was consistent across regions while influenza season duration varied more.

Understanding the annual activity and regional patterns of virus circulation has implications for the timing of respiratory hygiene campaigns, influenza vaccination, palivizumab, empiric antiviral treatment, and respiratory virus surveillance platforms. Similar to other countries,^27^ influenza activity in Argentina starts in the region with the country's capital and major air and maritime ports and, within 3 weeks, propagates to neighboring regions. Argentina vaccinates against influenza starting in March,^18^ and vaccines are offered throughout the influenza season.^19^ Influenza vaccine–induced immunity is thought to last through a season, but there is some evidence that protection wanes over time, especially for influenza A (H3N2) viruses.^28^ If Argentina's typical influenza annual activity starts no earlier than week 21 (mid‐May) and lasts up to October, delaying influenza vaccination campaigns until April when Vaccination Week of the Americas occurs could be advantageous.^24^ On the other hand, delaying vaccination may cause missed opportunities for vaccination, especially in the event of an early season. Vaccination programs need to take into account the unpredictability of influenza circulation. Any policy change in influenza vaccine timing could be coupled with communication campaigns to improve influenza vaccine coverage.^29^ In addition, region‐specific knowledge of RSV activity is important to determine when to administer RSV‐specific interventions such as palivizumab, as well as for any future vaccine and monoclonal antibody products in development.^30^

Public health officials in Argentina post timely surveillance updates on websites, but health advisories directed to more local community providers may also be beneficial, specifically outlining when influenza cases have been identified, how to triage severe respiratory infections, and whom to treat with empiric antivirals. Such reminders might be particularly important in Argentina where, even during the 2009 influenza pandemic, only 13% of children received antivirals within 48 hours of symptom onset.^31^ The cocirculation of RSV and influenza also has important implications for public health officials modeling the etiologic burden of disease in Argentina; because these viruses cocirculate, models must include robust age‐specific virologic data to meaningfully attribute clinical syndromes like pneumonia to RSV and/or influenza infection.^32^, ^33^

Certain limitations of these analyses should be considered. First, the number of specimens tested dropped in 2011 because of a transition from paper to electronic records. While Argentina tests a prodigious number of specimens a year for respiratory viruses, most are collected from hospitalized children in the capital^34^; therefore, the annual virus activity presented may be less representative of milder respiratory illnesses in areas outside of Buenos Aires and among adults. In general, influenza hospitalizations lag behind outpatient illness, but this depends on circulating influenza types and subtypes.^8^ Furthermore, two‐thirds of all respiratory specimens analyzed were collected from central Argentina where approximately 32% of the country's population lives; therefore, national annual virus activity may be overrepresented by central Argentina. Of note, the city of Buenos Aires is the main port of entry for people traveling to Argentina via air; therefore, robust specimen collection in the central region is expected and needed to efficiently identify representative respiratory virus circulation. Only one of 10 specimens submitted to ANLIS came from adults; this may explain the considerably higher RSV positivity than influenza positivity observed. Although it is possible that RSV and influenza testing practices could vary by region, the collection site and age of patients suspected of RSV and influenza did not differ greatly. While limitations in the age‐group and geographic representativeness of virologic surveillance do not substantially affect our analysis, it has important implications to Argentina's ability to rapidly detect an unusual surge in influenza virus in certain demographics (eg, among young adults as happened during the 2009 pandemic^35^). Given these limitations, expanding the geographic scope of influenza surveillance and increased respiratory testing among adults might be advantageous.

## 5 CONCLUSIONS

Respiratory syncytial virus and influenza cocirculate during the winter months in Argentina and follow a seasonal pattern that starts as early as April and lasts until October. Risk communication messages promoting handwashing, respiratory hygiene, influenza vaccination, palivizumab, antiviral treatment, and guidance as to when to stay home or seek care might be useful if launched as early as April each year. While there was little variability in the onset of influenza seasons in Argentina's geographically diverse regions, the start of RSV seasons varied within Argentina by region and by year. The duration of the influenza seasons also varied year‐to‐year. Continued monitoring of RSV and influenza is valuable, and Argentina's national health surveillance system may benefit from increasing collection in less densely populated regions of the country and among adults to improve representativeness. The distribution of public health advisories to clinicians outlining local RSV and influenza activity to better guide the timing of empiric antivirals and palivizumab immunoprophylaxis may be useful. Argentina starts vaccinating against influenza as early as March, but influenza activity typically does not start until June. It might be valuable to explore the potential benefits and risks of delaying the start of influenza vaccination to April during Vaccination Week of the Americas when PAHO and ministries of health promote immunizations.

## DISCLAIMER

The findings and conclusions in this report are those of the authors and do not necessarily represent the view of the CDC.
