# Supplementary material for: Timing of respiratory syncytial virus and influenza epidemic activity in five regions of Argentina, 2007‐2016
Source: Influenza Other Respir Viruses. 2018 Nov 20;13(1):10–7. doi: 10.1111/irv.12596 (PMC6304310; doi:10.1111/irv.12596)

Supplemental Figure 1. Annual national and regional respiratory syncytial virus activity in Argentina by epidemiological week, 2007–2013


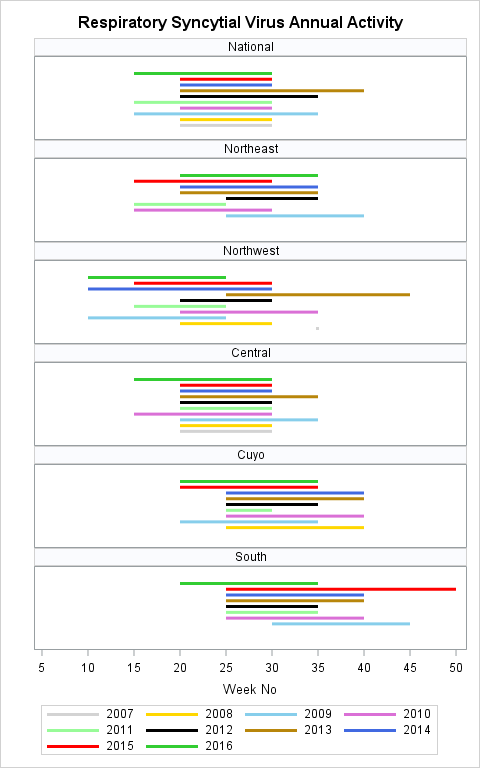


Supplemental Figure 2. Annual national and regional influenza activity in Argentina by epidemiological week, 2007–2013


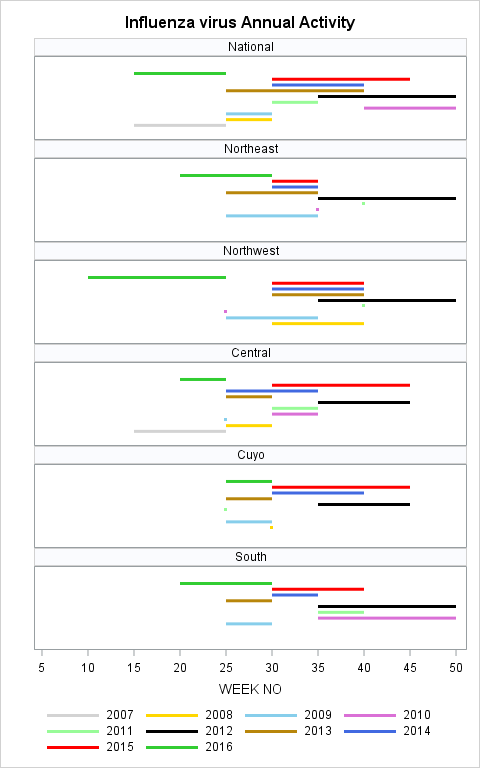

Supplement: Supplementary file 1 [file IRV-13-10-s001.docx]
